# Harmonizing population health data into OMOP common data model: a demonstration using COVID-19 sero-surveillance data from Nairobi Urban Health and Demographic Surveillance System

**DOI:** 10.3389/fdgth.2025.1423621

**Published:** 2025-01-28

**Authors:** Michael Ochola, Sylvia Kiwuwa-Muyingo, Tathagata Bhattacharjee, David Amadi, Maureen Ng’etich, Damazo Kadengye, Henry Owoko, Boniface Igumba, Jay Greenfield, Jim Todd, Agnes Kiragga

**Affiliations:** ^1^ Data Science Program, African Population and Health Research Center (APHRC), Nairobi, Kenya; ^2^ Department of Population Health, Faculty of Epidemiology and Population Health, London School of Hygiene and Tropical Medicine, University of London, London, United Kingdom; ^3^ Machine Learning (AI and ML), Committee on Data of the International Science Council (CODATA), Paris, France; ^4^ Implementation Network for Sharing Population Information from Research Entities (INSPIRE Network), Nairobi, Kenya

**Keywords:** observational health data, OMOP CDM, population health data, COVID-19, sero-surveillance, Nairobi Urban HDSS, ETL, Africa

## Abstract

**Background:**

Observational health data are collected in different formats and structures, making it challenging to analyze with common tools. The Observational Medical Outcome Partnership (OMOP) Common Data Model (CDM) is a standardized data model that can harmonize observational health data.

**Objective:**

This paper demonstrates the use of the OMOP CDM to harmonize COVID-19 sero-surveillance data from the Nairobi Urban Health and Demographic Surveillance System (HDSS).

**Methods:**

In this study, we extracted data from the Nairobi Urban HDSS COVID-19 sero-surveillance database and mapped it to the OMOP CDM. We used open-source Observational Health Data Sciences and Informatics (OHDSI) tools like WhiteRabbit, RabbitInAHat, and USAGI. The steps included data profiling (scanning), mapping the vocabularies using the offline USAGI and online ATHENA, and designing the extract, transform, and load (ETL) process using RabbitInAHat. The ETL process was implemented using Pentaho Data Integration community edition software and structured query language (SQL). The target OMOP CDM can now be used to analyze the prevalence of COVID-19 antibodies in the Nairobi Urban HDSS population.

**Results:**

We successfully mapped the Nairobi Urban HDSS COVID-19 sero-surveillance data to the OMOP CDM. The standardized dataset included information on demographics, COVID-19 symptoms, vaccination, and COVID-19 antibody test results.

**Conclusions:**

The OMOP CDM is a valuable tool for harmonizing observational health data. Using the OMOP CDM facilitates the sharing and analysis of observational health data, leading to a better understanding of disease conditions and trends and improving evidence-based population health strategies.

## 1 Introduction

Population health studies play a key role in understanding specific populations or groups’ health status, determinants, and outcomes. Analyzing data from different sources is challenging due to inconsistencies in structures, formats, definitions, standards, coding systems, and database platforms. Data harmonization is a key process that addresses these challenges by standardizing and transforming data to enable seamless integration and enhanced interoperability and ensure consistency across diverse datasets for more accurate analysis, informed decision-making, and a unified understanding of information across different systems for meaningful comparisons and analyses. The extract, transform, and load (ETL) pipeline is the process embedded into the workflow, which does the standardization and transformation tasks to put data in a common standard, referred to as a common data model.

Population health data often include various indicators beyond clinical records, such as socioeconomic factors, and environmental and lifestyle data. The data may need to be aggregated, and granularity of data such as community-level factors and integrating population health outcomes are not comprehensively addressed in traditional clinical data for which OMOP CDM was intended. Adapting this to OMOP CDM to incorporate diverse data types introduces complexities. Extension to the OMOP Common Data Model is necessary to address these challenges, and interoperability with other data standards and models used in population health research must be carefully considered. Data harmonization is vital in population health studies. It improves data quality (1), enhances comparability across studies (2, 3), and facilitates large-scale analyses. This paper provides insights into the data harmonization process essential for transforming data from a COVID-19 Sero-surveillance at Nairobi Urban Health and Demographic Surveillance (NUHDSS) area into Observational Medical Outcomes Partnership (OMOP) Common Data Model (CDM), which is an open community data standard, intended to standardize the structure, format, and content of observational clinical data and to enable practical and effective analyses that can produce consistent evidence for better policymaking. This paper highlights the vital re-orientation process performed to adopt and use the clinical-based OMOP CDM in population studies.

### 1.1 Observational health data sciences and informatics (OHDSI)

The availability of medical records, surveys, population surveillance data, and other real-world data from the field of medical care and public health systems gives many opportunities for research and evidence-based understanding. However, utilizing the potential of such vast data requires standardized methods, collaborative efforts, and easy-to-access analytical tools. The Observational Health Data Sciences and Informatics (OHDSI) addresses many of these challenges and assists in large-scale analytics of observational health data. OHDSI is an international, multi-stakeholder, interdisciplinary, open-source collaborative that aims to improve health by empowering a community to collaboratively generate evidence that promotes better health decisions and better care (4).

OHDSI focuses on open science by promoting transparency and reproducibility in research. Specifically, OHDSI supports the development of open-source software tools for the community. The Observational Medical Outcomes Partnership (OMOP) Common Data Model (CDM) is one such effort, which facilitates the harmonization of heterogeneous health data using standardized terminologies and common data models. The common data model and standardized vocabularies set the stage for uniform analytics (5, 6).

To achieve the common data structure and leverage the use of big data analytics tools, building ETL (extract, transform, and load) is necessary for extracting source data, transforming it into a standard format of the OMOP CDM data structure, and loading it into the OMOP CDM database (7). The extract, transform, and load process are a series of tasks designed to align source data with the structure and terminology of the target database. Supporting data harmonization typically involves two consecutive phases, each handled by professionals with distinct expertise. During the first phase, subject matter experts familiar with the source data (e.g., EHR, claims, population health and mental health data) identify the necessary data elements for extraction and define mappings between the source and target data elements. This step demands expertise in both source and target data, encompassing domain knowledge of local population health implementations and terminologies. In the second phase, database programmers execute data transformation methods and schema mappings to load data into the OMOP CDM-ready tables.

### 1.2 The source data

The study utilized archived secondary data from the African Population and Health Research Center (APHRC) online microdata portal (https://microdataportal.aphrc.org/index.php/catalog/138), it provides metadata and a high-level overview of various research outputs within the center, datasets can be accessible for further research by requesting the portal managers. Data were collected within Nairobi Urban Health and Demographic Surveillance Systems (NUHDSS). The NUHDSS is a pioneer in urban-based HDSS sites. It was created to fill an evidence gap in understanding the challenges stemming from rapid urbanization, the growing concentration of the urban poor, and health status within two informal settlements (Korogocho and Viwandani) in 2002 (8).

The data was collected as part of a COVID-19 seroprevalence survey within representative samples of the Kenyan population approximately two years into the COVID-19 pandemic and approximately one year after the rollout of the national COVID-19 vaccination program (9).

The primary sero-surveillance study sample comprised 870 individuals randomly selected from the NUHDSS database, reflecting diverse socio-demographic characteristics, from whom the blood samples were collected to ascertain the presence or absence of COVID-19 antibodies. In total, the dataset had approximately 140 variables, and a subset (60%) of these variables have been harmonized into OMOP CDM based on the success of source-to-target mapping i.e., from the source codes to OMOP CDM vocabularies using the ATHENA vocabulary repository, and the availability of concepts in respective vocabulary sets.

The dataset was anonymized before uploading into the micro-data portal in adherence with data protection guidelines. Additionally cleaning, quality checks, and descriptive statistical analysis were performed before the start of the actual harmonization pipeline as shown on Table 1. It is important to highlight that during the initial implementation of sero-surveillance at the primary data collection stage, the researchers adhered to ethical standards and approvals as expected by Kenya's research governing body (10).

**Table 1:** Overview of NUHDSS COVID-19 sero-survey data scan report.

Table	Field	Description	Type	Fraction empty	*N* unique values
sero_results.csv	Studyid	Sero study id	INT	0.0%	870
sero_results.csv	q8_gender	Individual q8_gender	VARCHAR	0.0%	2
sero_results.csv	q7_birth_date	Individual's date of birth	VARCHAR	0.0%	745
sero_results.csv	q7_age	q7_age in complete years	INT	0.0%	82
sero_results.csv	Age_strata	Individual's q7_age strata	VARCHAR	0.0%	15
sero_results.csv	q9_location	Individual location	VARCHAR	0.0%	2
sero_results.csv	q5_status	Individual q5_status	VARCHAR	0.0%	1
sero_results.csv	Consent	Individual consented	VARCHAR	0.0%	1
sero_results.csv	Replace	Individual replaced	VARCHAR	0.0%	2
sero_results.csv	q6_education_level	School level	VARCHAR	0.0%	4
sero_results.csv	q6_religion	Religion	VARCHAR	0.0%	5
sero_results.csv	q10_covid_contact	Suspected or confirmed COVID-19	VARCHAR	0.0%	4
sero_results.csv	q10_contact_month	Contact month	VARCHAR	0.0%	11
sero_results.csv	q10_contact_year	Contact year	VARCHAR	0.0%	3
sero_results.csv	q11_fever	History of fever/chills	VARCHAR	0.0%	2
sero_results.csv	q11_shortness_breath	Shortness of breath	VARCHAR	0.0%	2
sero_results.csv	q11_pain	Pain	VARCHAR	0.0%	2
sero_results.csv	q11_weakness	General weakness	VARCHAR	0.0%	2
sero_results.csv	q11_diarrhoea	Diarrhoea	VARCHAR	0.0%	2
sero_results.csv	q11_cough	Cough	VARCHAR	0.0%	2
sero_results.csv	…	…	…	…	…
sero_results.csv	Spikepos	Antibodies present in sample	VARCHAR	0.1%	3
sero_results.csv	Latitude	Latitude	REAL	0.0%	2
sero_results.csv	Longitude	Longitude	REAL	0.0%	2

With a conventional basic descriptive analysis of the data. It showed the majority of study participants were under the age of 35, which is typical of informal settlements, with males slightly leading. Overall, nearly half of the respondents had completed primary school as their highest level of education as shown on Table 2.

**Table 2:** Sero-prevalence by various demographics characteristics.

Age categories	Sero-negative	Sero-positive	Total
	*N* = 539	*N* = 330	*N* = 869
less than 5	85 (15.77)	18 (5.45)	103 (11.84)
5–9 years	86 (15.96)	25 (7.58)	111 (12.76)
10–14 years	65 (12.06)	38 (11.52)	103 (11.84)
15–19 years	22 (4.08)	32 (9.7)	54 (6.21)
20–24 years	36 (6.68)	17 (5.15)	53 (6.09)
25–29 years	31 (5.75)	15 (4.55)	46 (5.29)
30–34 years	27 (5.01)	18 (5.45)	45 (5.17)
35–39 years	25 (4.64)	27 (8.18)	52 (5.98)
40–44 years	25 (4.64)	25 (7.58)	50 (5.75)
45–49 years	27 (5.01)	23 (6.97)	50 (5.75)
50–54 years	29 (5.38)	18 (5.45)	47 (5.4)
55–59 years	26 (4.82)	27 (8.18)	53 (6.09)
60–64 years	33 (6.12)	18 (5.45)	51 (5.86)
65 years and above	22 (4.08)	29 (8.79)	51 (5.86)
Gender			
Male	285 (52.88)	173 (52.42)	458 (52.70)
Female	254 (47.12)	157 (47.58)	411 (47.30)
Location			
Korogocho	216 (40.07)	120 (36.36)	336 (38.67)
Viwandani	323 (59.93)	210 (63.64)	533 (61.33)
Education			
No formal education	143 (26.53)	53 (16.06)	196 (22.55)
Primary	254 (47.12)	168 (50.91)	422 (48.56)
Secondary	121 (22.45)	93 (28.18)	214 (24.63)
Tertiary	21 (3.9)	16 (4.85)	37 (4.26)

## 2 Methods

### 2.1 Designing the data harmonization workflow using OHDSI tools

**Figure 1:**
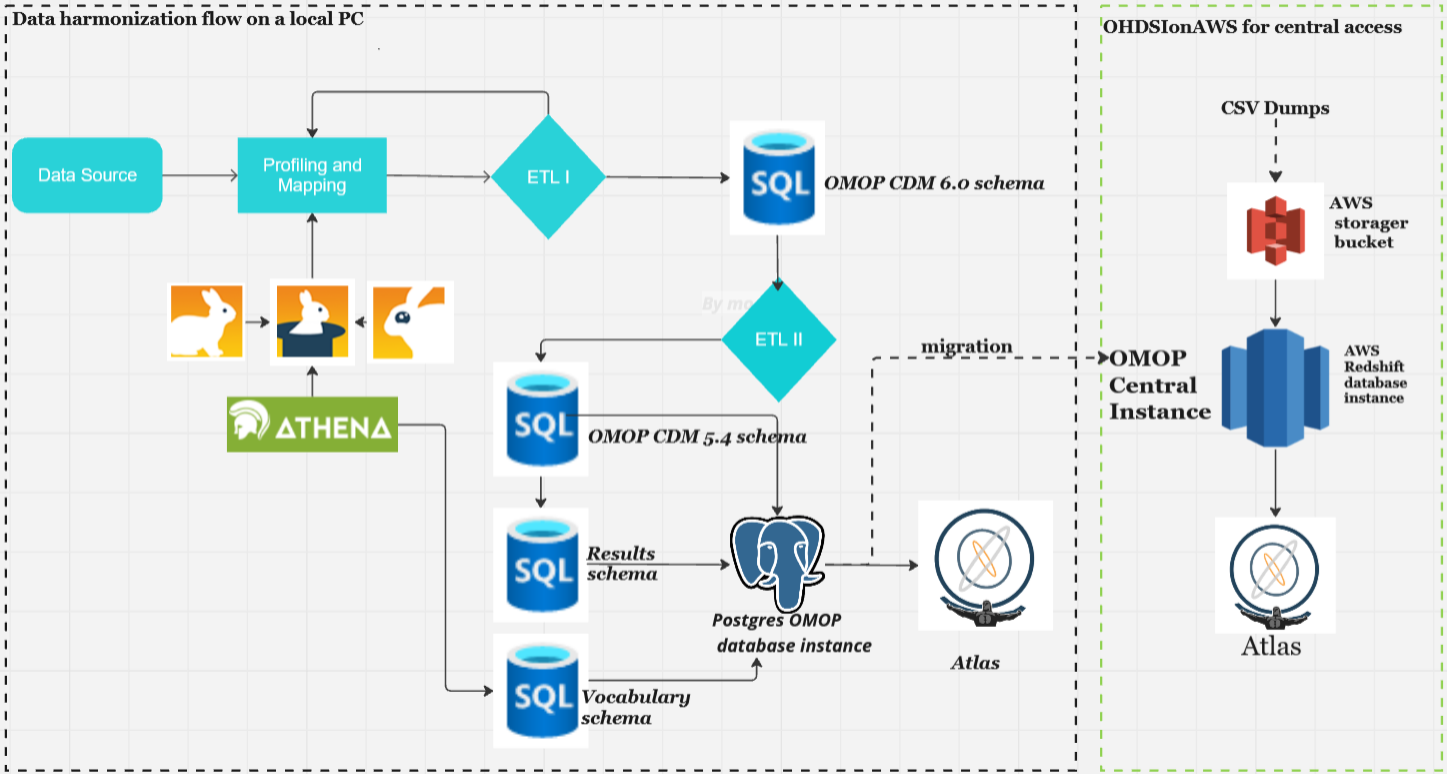
Harmonization pipeline architecture for NUHDSS COVID-19 sero-survey.

Figure 1 shows data harmonization and integration workflows for the NUHDSS data. The first step in the data harmonization pipeline was an exploratory data analysis (EDA). In the OHDSI suite of tools, WhiteRabbit performs scanning and profiling of the source data. This stage yielded a comprehensive overview of the source data content, encompassing details like data types, occurrences of missing data, data dimension, and summary statistics of numeric variables. WhiteRabbit accommodates various data sources, spanning from flat files to relational databases. As a result, seamless integration with any source data is facilitated (11). EDA was followed by “mapping”, also referred to as ETL design. During mapping a scan report produced by WhiteRabbit was uploaded into Rabbit-in-a-Hat. This tool is Java-based and platform-independent, serving as a valuable aid in the creation of the ETL specification document. This step builds upon the implementation guide already generated by the USAGI tool, which mapped source codes to the standard OMOP CDM codes (12–14).

**Figure 2:**
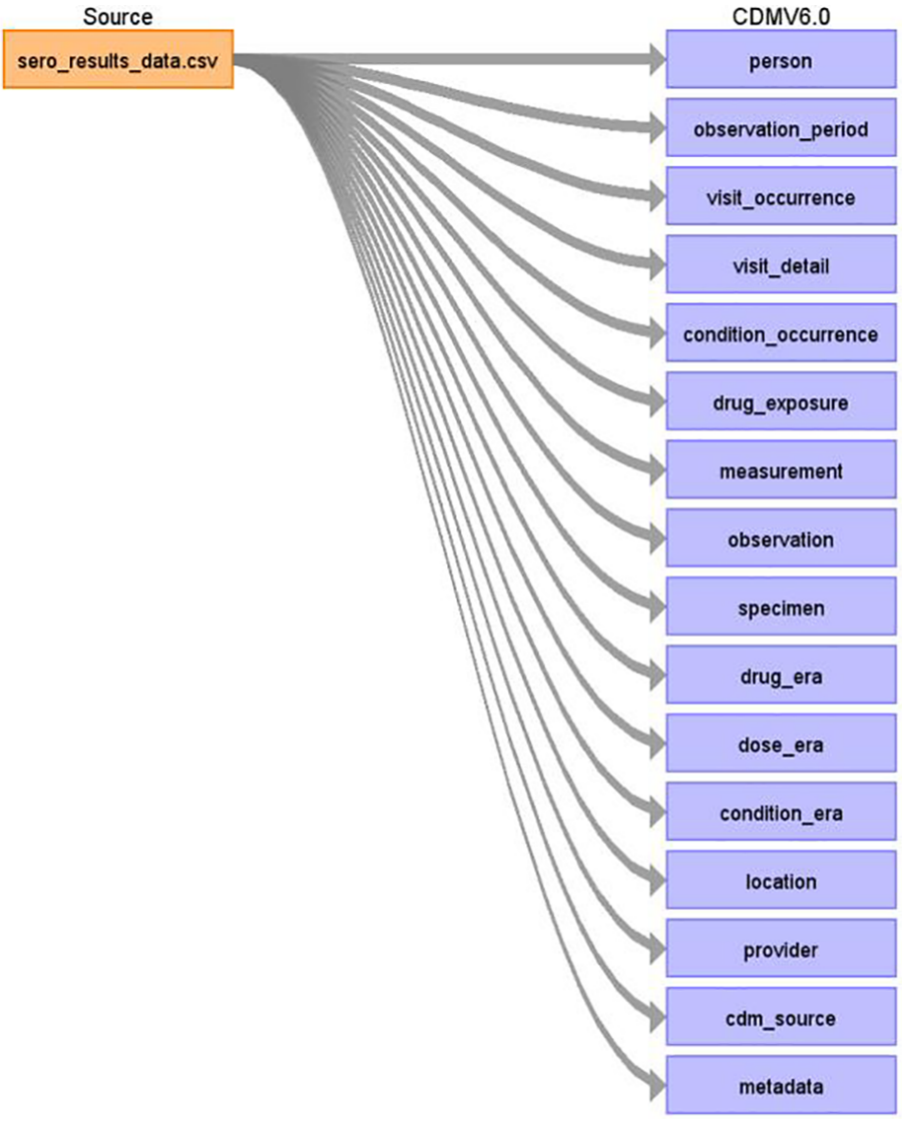
High-level overview of NUHDSS COVID-19 sero-survey data mapped to OMOP CDM tables.

Figure 2 shows a detailed mapping of the source data to OMOP CDM schema version 6.0 respective tables as defined by the source data variables. Some of the notable tables mapped include person, observation_period, visit_occurrence, visit_detail, condition_occurrence, drug exposure, measurement, observation, specimen, drug_era, dose_era, condition_era, location, provider, cdm_source, and metadata (15).

**Figure 3:**
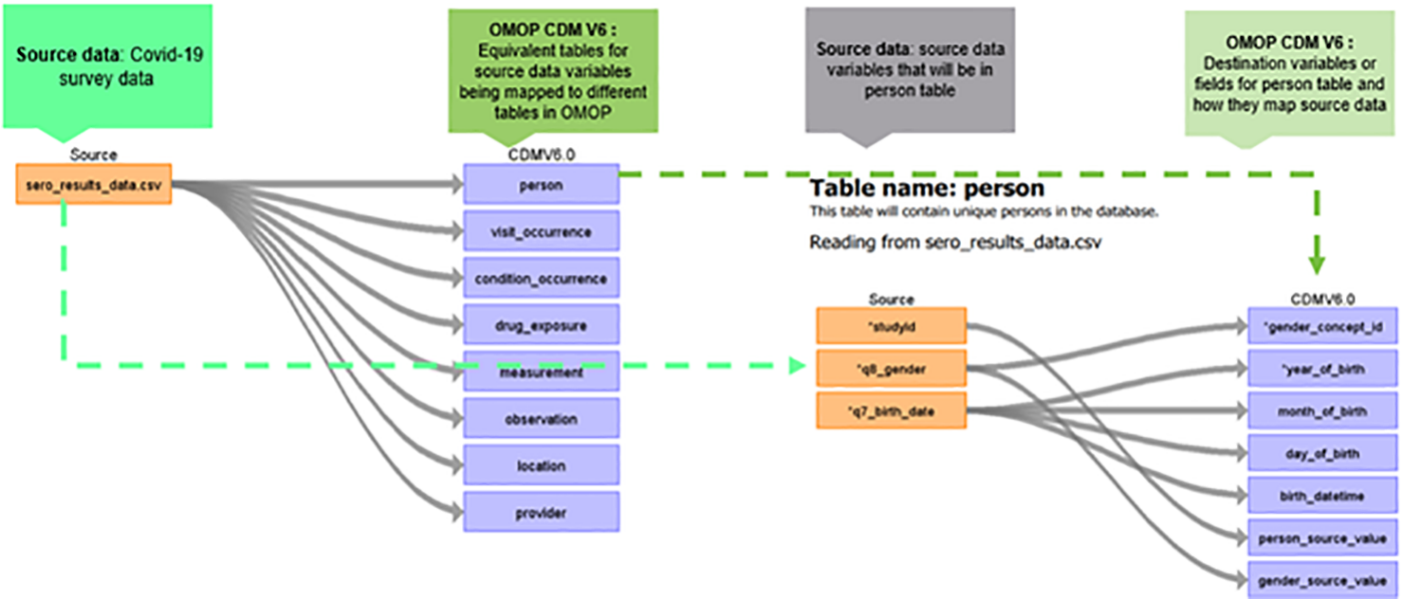
Mapping from source data to OMOP CDM person table.

It is best practice to anonymize data before harmonization. As stated earlier, sero-survey data was anonymized. During the anonymization, key personal identifiers had been removed from the data prior to uploading on the APHRC microdata portal. During mapping, the first table mapped to the OMOP CDM was the person table which contained information regarding the patient's gender, date of birth, ethnicity, race, and other variables like location code, provider code, and care_site code obtained through linkage with other tables within the OMOP CDM (16). Figure 3 shows how a subset of sero-survey data was mapped to the person table. Study_id or row unique identifier in the source data was mapped to person_source_value, gender was mapped to gender_concept_id which has standardized codes for both males and females. Lastly, the birth_date variable was used to generate within the OMOP CDM person table year_of_birth, month_of_birth and day_of_birth.

Figure 3 describes the mapping from the source to the OMOP CDM's person table. This was an important first step in mapping as the study participants’ demographic details are populated into this table.

The third stage involved downloading the OHDSI vocabulary into the local database instance. ATHENA, an online OHDSI vocabulary repository (12), was utilized to incorporate the current vocabulary version in preparation for ETL development. This process involved populating a vocabulary schema in a PostgreSQL database from the OHDSI ATHENA repository. The applicable standard vocabularies were obtained as comma-separated (csv) files and then loaded into the corresponding tables within the vocabulary schema using the SQL COPY command. Typically, this is a one-time task, unless there are updates to the ATHENA vocabularies. The mapping process generated an ETL specification document used for ETL development.

**Figure 4:**
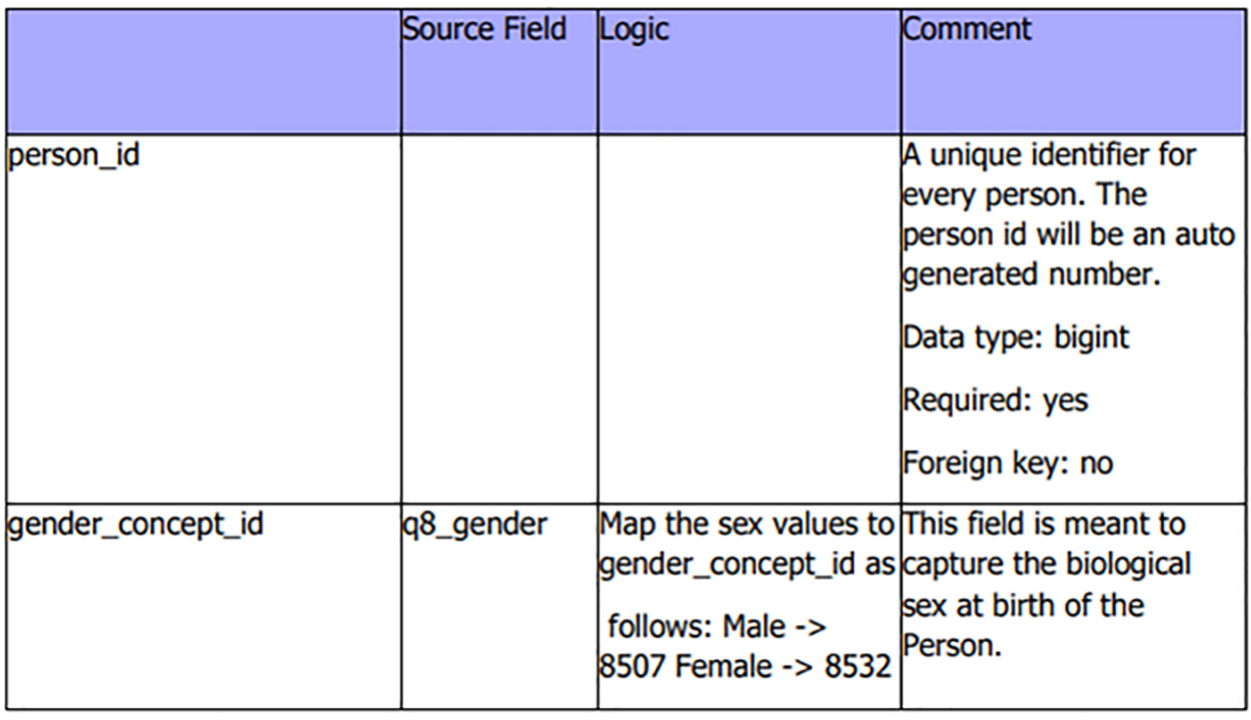
ETL specification document extract from person table with some variables.

Figure 4 is an extract of an ETL specification document for the person table depicting the code-level mappings. The process above was replicated for all the tables, and the resulting ETL specification document was passed on for actual ETL development.

The mapping process for sero-survey data to the OMOP CDM schema encountered some challenges. Some variables representing vaccines given to young children and socioeconomic status (SES) lacked clearly defined standard codes or concepts within the OHDSI vocabulary and, therefore, were mapped to concept code zero as the OHDSI standard for missing concepts. This problem is not unique to this dataset mainly because concepts primarily developed to annotate clinical data in Europe and America will need localization when it comes to population health data collected on the African content (17).

An ETL process involves combining data from multiple sources into a centralized database (18). Data is extracted from different sources and stored on a temporary schema. A transform step will then consolidate the raw data in the temporary schema to prepare it for the OMOP CDM respective tables, either through data deduplication, data format revisions to match target schema, splitting, cleansing, encryption, and joining data. Lastly, in the loading step, transformed data is moved from the staging schema to the target database schema. ETL uses a set of rules to clean and structure the raw data, making it ready for data analysis and machine learning applications (19).

Before the actual ETLs were executed on the survey data, additional exploratory data analysis (EDA) was conducted to understand the demographics of the source data. This proved useful during the quality checks on the final ETL, where both EDAs were compared. The actual ETL workflow for the NUHDSS COVID-19 sero-survey started with data extraction. Here the archived data from the micro-data portal was read into an R program.

R is a programming language and environment specifically designed for statistical computing and graphics. It provides a wide variety of statistical and graphical techniques, making it a popular choice for data analysis and visualization. The data was converted into comma-separated values for ease of use within the ETL suite of tools. Pentaho data integration (PDI) and structured query language (SQL) were used to orchestrate the workflows that took the csv file and pipelined it to the OMOP CDM ready tables. Pentaho Data Integration, commonly known as PDI and previously referred to as Kettle, is an ETL tool encompassing a suite of software applications tailored for creating data workflows. These workflows can be executed either within server environments or as standalone processes. PDI is characterized by two primary components: Kitchen, serving as a runner for jobs and transformations, and Spoon, a graphical user interface specifically designed for the creation and configuration of these jobs and transformations (20). With SQL all the OMOP CDM table structures are generated using already available table creation scripts provided by the OHDSI community, covering at least major database engines both for on-premises and cloud platforms (21, 22). In addition, the source dataset schema is created mainly to serve as a staging database.

**Figure 5:**
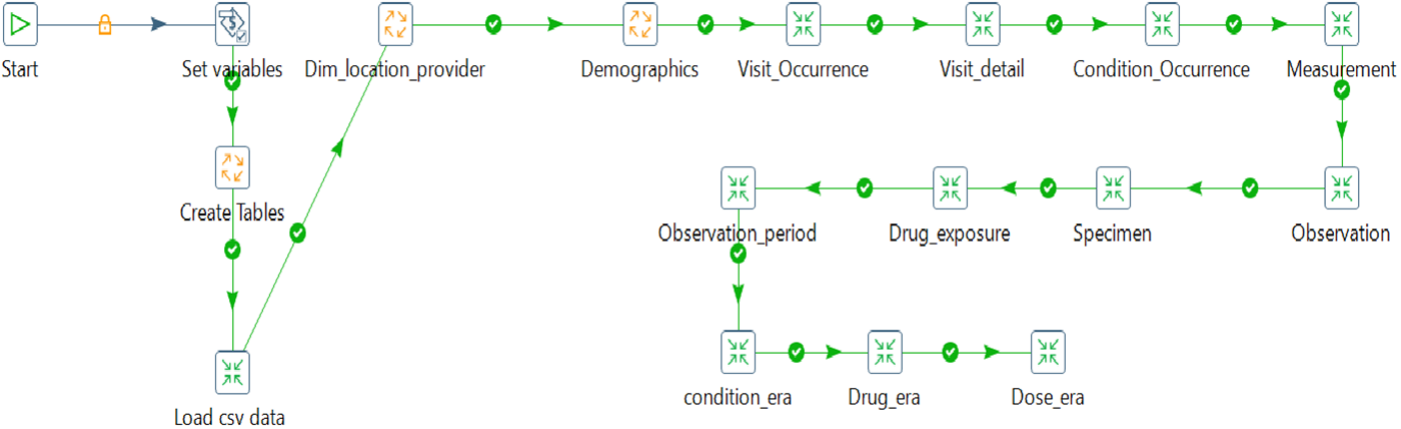
Pentaho ETL pipeline with various transformations and jobs moving data to OMOP CDM v6.0.

So, the source csv data was read into a table created within the staging schema. Transformation of data in the staging database using the SQL scripts and a combination of PDI transforms and jobs created table structures in alignment with the respective OMOP CDM tables. The provider, location, and care site tables were generated and loaded, followed by a person, visit occurrence, visit detail, condition occurrence, measurement, observation, specimen, drug exposure, observation_period, condition_era, drug_era, and dose_era as shown on Figure 5. PostgreSQL was used here as the target database engine, although OMOP CDM supports several database engines, therefore promoting interoperability as an integral component of the Findable Accessible Interoperable, and Reusable (FAIR) principle in data systems.

Harmonized data within the OMOP CDM version 6 was migrated to CDM version 5.4 to allow for backward compatibility with the OHDSI suite of tools. The migration involved loading data from OMOP CDM 6.0 tables to respective OMOP 5.4 tables, which required either data type alignment, dropping column(s), and removing the required attribute on the date and datetime fields. This was necessary to allow usage of the OHDSI suite of tools currently supporting OMOP CDM versions 5.4 and 5.3. The data harmonization pipeline, along with detailed documentation of the process, is available on GitHub (https://github.com/APHRC-DSE/Sero-data-to-OMOP-ETL).

### 2.2 Data quality checks

After a successful ETL process, quality assurance of the resulting common data model was undertaken. A team verified an ETL design document to ensure that data was mapped to the appropriate OMOP CDM tables and fields. This verification resulted in documentation of the design phase. Next, SQL scripts, Pentaho transformations, OMOP CDM tables, and the data were verified for any mistakes or incorrect mappings.

After harmonization, data quality metrics were produced. They encompassed various aspects, including the comparison of the total number of individuals in the OMOP CDM person table with the source data, and the same number of distinct individuals reported in exposure and era tables. The last quality check on the harmonized data was implemented using the OHDSI suite of tools; ACHILLES and data quality dashboard (DQD) (23). DQD is an open-source R library supported in OMOP CDM versions 5.4, 5.3, and 5.2. It runs a series of systematic checks on the OMOP CDMready database instance and supports multiple relational database management systems (RDBMS) including PostgreSQL, Microsoft SQL server, Google Big Query and Amazon Redshift popular for cloud deployment using the OHDSI on cloud architecture. The tool applied over 3300 quality checks on the OMOP CDM database, grouped into conformance, completeness, and plausibility checks. For conformance checks the aim was to ensure data adhered to the specified format and standards. Completeness checked if data values were present, and plausibility checked if a given measurement and a given unit based on context were valid. Notable errors checked were: any records after death date, persons lacking recorded year of birth, missing gender, and more. The approach to correct the identified quality issues involved examining the source data or ETL pipeline to determine the appropriate fix (24–26).

Data analysis on OMOP CDM-ready tables took three forms. First, the OHDSI ACHILLES R library was used to characterize the data. Secondly, OHDSI ATLAS, a low code data analysis workbench, was used to produce descriptive statistics on persons and various symptom distributions. Thirdly, specialized libraries and analysis packages tailored to individual OMOP CDM instances expands the range of possibilities for developing custom algorithms for descriptive and inferential analysis. In this study, we used a combination of these approaches (27).

## 3 Results

As previously specified, a quality assessment for the process and the output was carried out using the data quality dashboard (DQD), this provided a mechanism to check the resulting OMOP CDM for conformance, completeness, and plausibility, and set the stage for ATLAS visualization. Data quality assessment through the dashboard in Table 3 showed the resultant quality of the ETL process that generated OMOP CDM data had a 98% quality score. Additional quality checks to ensure data reliability and consistency were carried out, cross-referencing OMOP CDM aggregates against raw data aggregates from the EDA.

**Table 3:** Data quality dashboard for harmonized COVID-19 sero-survey.

	Verification				Validation				Total			
	Pass	Fail	Total	% Pass	Pass	Fail	Total	% Pass	Pass	Fail	Total	% Pass
Plausibility	2,171	0	2,171	100%	287	0	287	100%	2,458	0	2,458	100%
Conformance	660	68	728	91%	106	0	106	100%	766	68	834	92%
Completeness	376	20	396	95%	15	2	15	88%	391	22	413	95%
Total	3,207	88	3,295	97%	408	2	408	100%	3,615	90	3,705	98%

**Figure 6:**
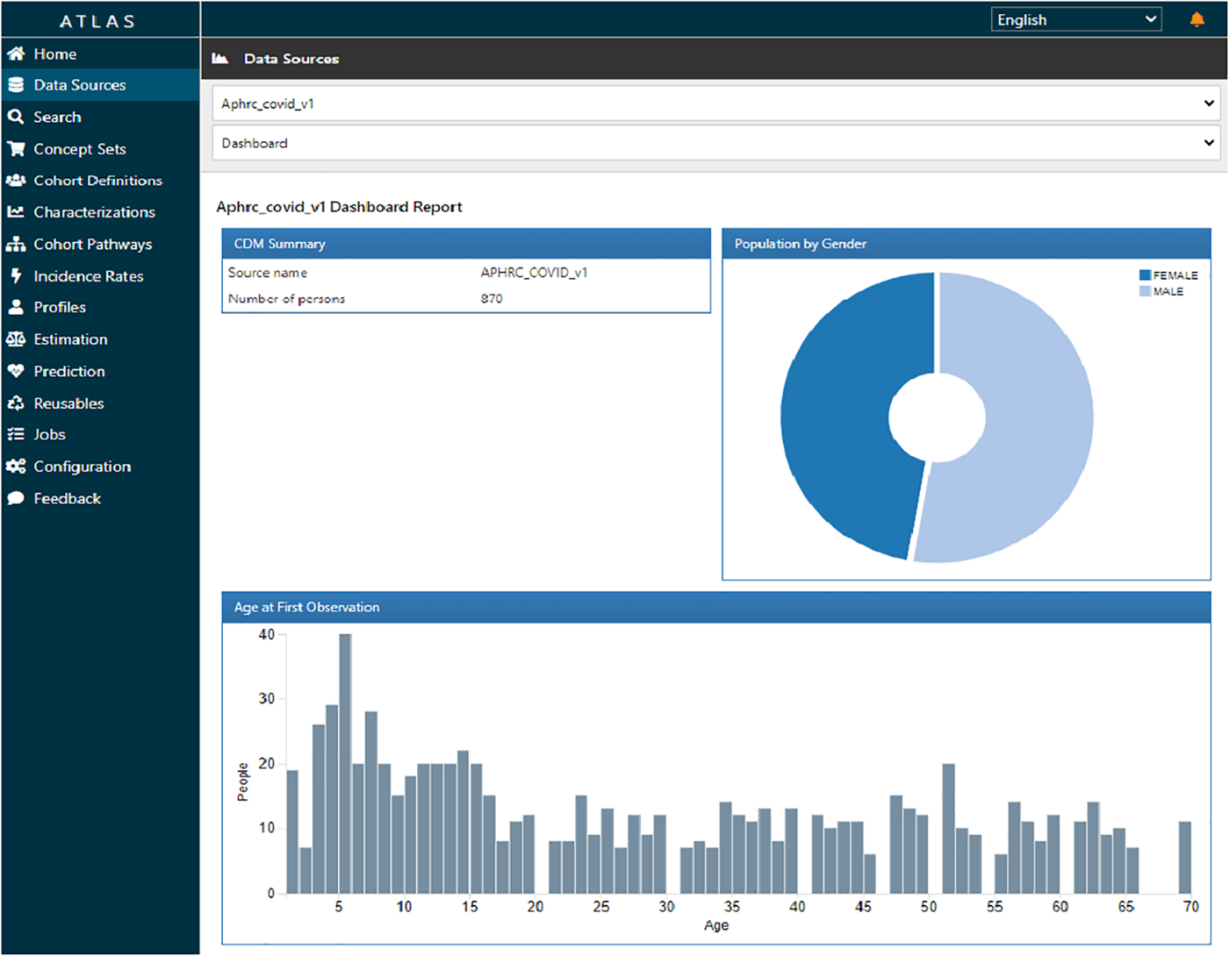
ATLAS dashboard report for harmonized COVID-19 sero-survey.

Figure 6 shows ATLAS dashboard reports and visualizations of the harmonized sero-survey data. We used the data sources module in ATLAS to explore database characteristics created by ACHILLES. The harmonized sero-survey data comprised 870 individuals, with 52% of respondents being males. The age distribution ranged from 0 to 70 years, with a predominant majority below 35 years, a characteristic common in most informal settlements.

**Figure 7:**
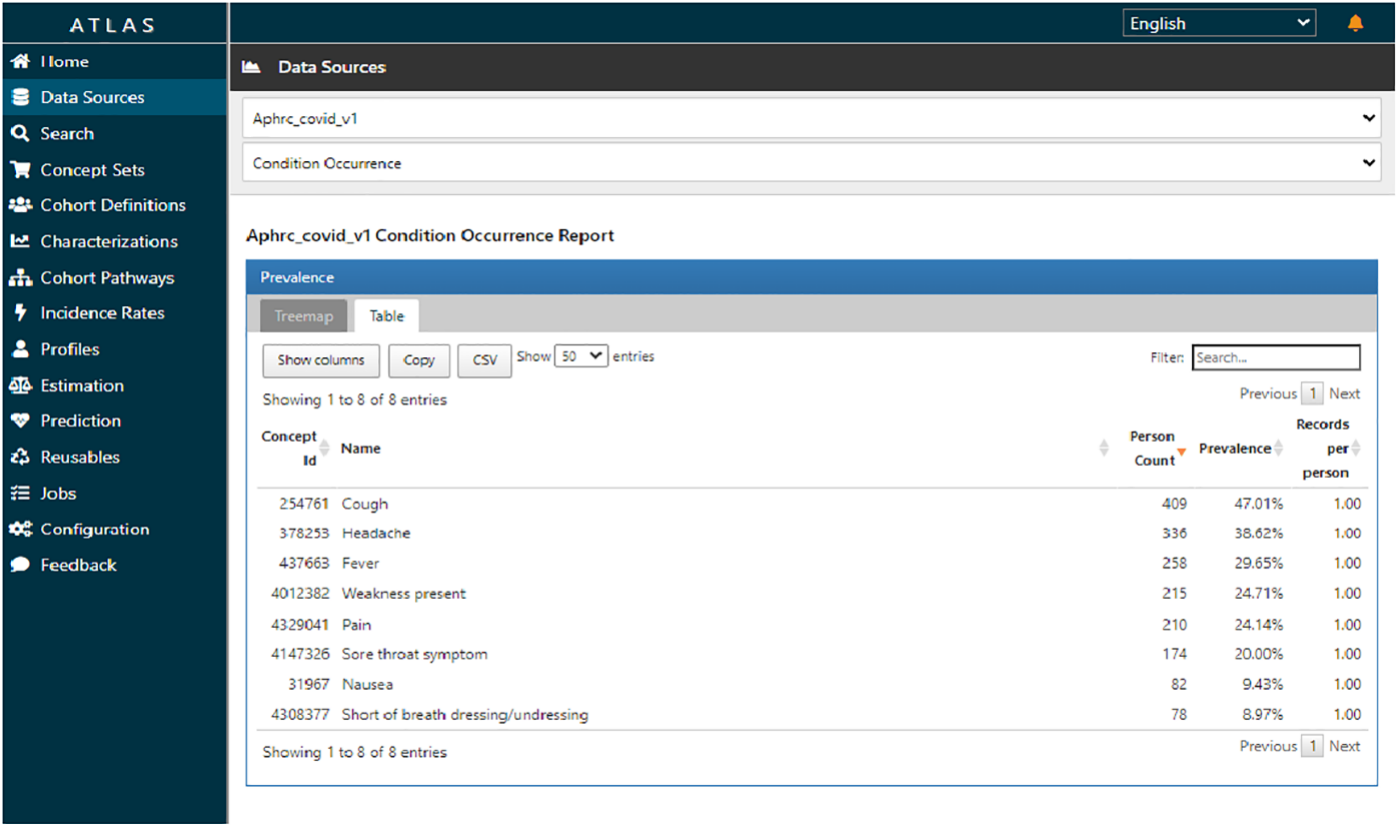
ATLAS condition occurrence report for harmonized COVID-19 sero-survey.

Figure 7 shows the prevalence of various conditions observed. Notably, a significant number of individuals experienced cough, headache, and fever with a single condition record per person interviewed, this being a cross-sectional study.

**Figure 8:**
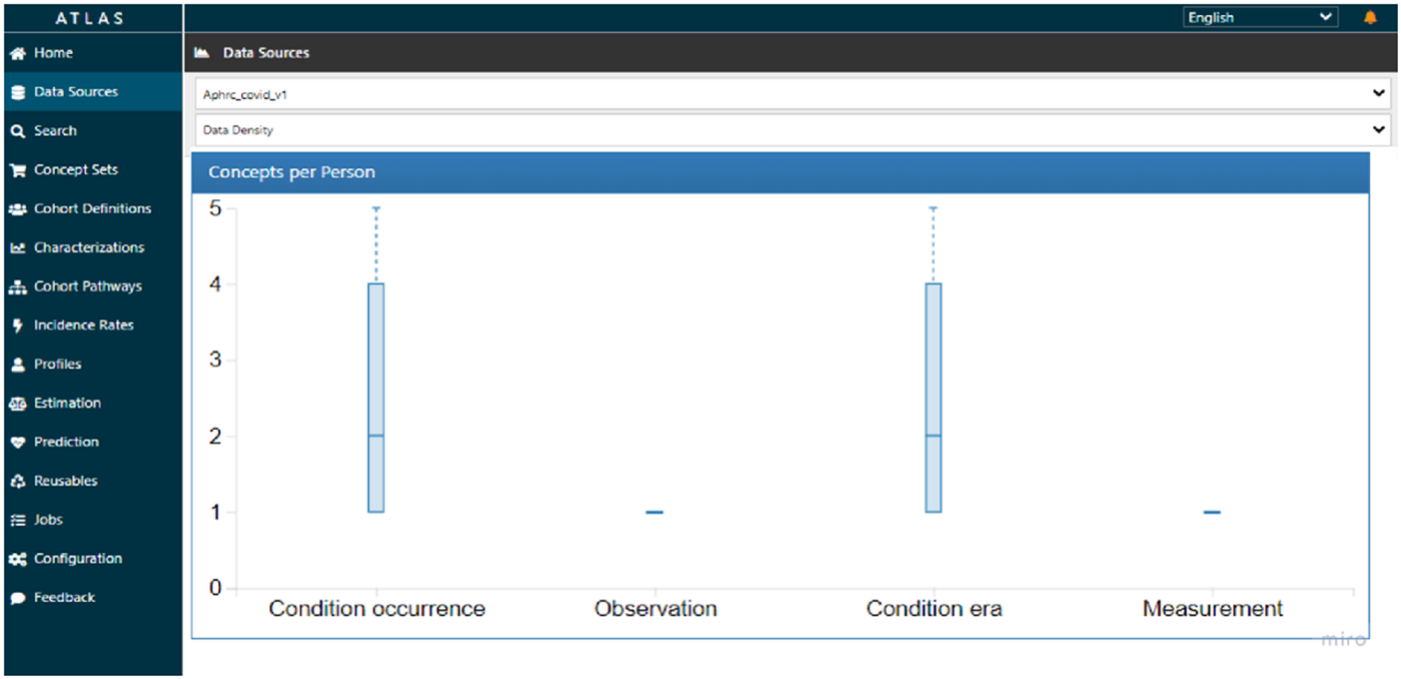
ATLAS data density plot on condition occurrence for harmonized COVID-19 sero-survey.

The median number of reported conditions was two, though some individuals reported as many as five conditions, as indicated on Figure 8. This figure suggests that it was likely to have a cough with headache or cough with fever, or any other combination. There was no variation to report on observation and measurement concepts for this data.

**Figure 9:**
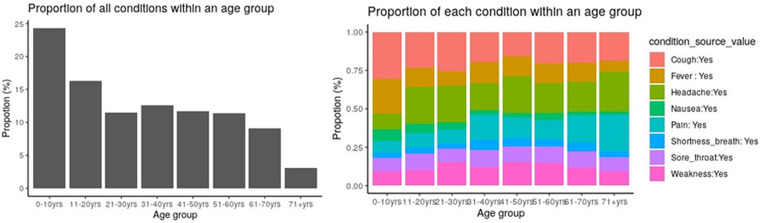
Proportion of conditions across age groups.

In addition to utilizing the ATLAS dashboard for visualization, OMOP CDM databases offer the flexibility of programmatic access through widely used data science scripting languages. R was integrated with the OMOP CDMdatabase instance to visualize the proportion of various conditions across age groups as shown in Figure 9.

The proportion of respondents who tested positive for SARS-CoV-2 antibodies was 39%. The analysis could also be achieved in ATLAS using its point-and-click interface to construct cohorts with particular feature sets. Notably, the analysis of COVID-19 vaccination was omitted due to insufficient data records pertaining to vaccination uptake in the available data.

## 4 Discussion

Utilizing the OMOP CDM for data harmonization offers numerous benefits, its uniqueness lies in its use of OHDSI vocabularies, which facilitate seamless interoperability with leading health sector vocabulary standards. As long as the necessary vocabularies are supported, studies can be integrated into the OMOP CDM framework. Expanding these vocabularies further extends the potential to harmonize data across a wide range of domains.

In our study, converting COVID-19 sero-surveillance data from the Nairobi Urban HDSS to the OMOP CDM facilitated the uniformity of data, simplifying subsequent analysis through available OHDSI tools and software packages. This harmonization process not only standardized the data but also enhanced its profiling and accessibility for researchers, establishing a shared format for data encoding, structure, and database software. Moreover, it enabled seamless data exploration and visualization through the user-friendly ATLAS interface.

The Data Quality Dashboard (DQD) results showed that the dataset's quality for the OMOP CDM was at 98%, meeting most of the quality checks built into the OHDSI DQD R package. This was the outcome of a successful data migration process from the source to the OMOP CDM: adopting the ETL approach for this migration resulted in high-quality output.

Integrating data into the OMOP CDM, however, comes with its share of challenges, with one of the primary concerns being the inability to completely map all data to a standardized vocabulary from the ATHENA repository. The vocabulary accessible through the ATHENA repository is a continually evolving collection that may lack definitions for numerous conditions across various contexts. These vocabularies are classified into different domains and sets, and they may not perfectly align with every aspect of the source dataset.

More specifically, some variables from the source dataset didn't fit well in the OMOP CDM standardized concepts. A few instances like the work missed due to the infection, some childhood vaccinations like the Penta & BCG along with the household levels variables from the social economic status questionnaire didn't map to the existing standardized concepts in the OMOP CDM. This results into a potential data loss on the final OMOP CDM, which could limit some downstream processes, including visualization, analysis, and modeling referencing such variables.

One viable solution to address this issue involves extending the existing sets by introducing new concepts tailored to different types of studies. The ATHENA vocabulary repository is structured in a hierarchical organization of concepts and classes. Therefore, adding new concepts must be implemented methodically to avoid disrupting this hierarchy. This hierarchy plays an important role in data analysis, especially when using tools like ACHILLES R scripts, and in visualizing the data through the ATLAS interface. In this regard, the Implementation Network for Sharing Population Information from Research Entities (INSPIRE) network is at the forefront of championing the development of vocabulary concepts for population health data within the vocabulary working group OHDSI Africa chapter.

Another challenge encountered during the mapping process from the APHRC COVID-19 sero-surveillance dataset pertained to the restricted availability of variables and values within the source data. This limitation posed hurdles in comprehensively representing and capturing the characteristics of the data, as certain essential variables and values were either absent or insufficiently documented. This challenge highlighted the importance of careful data collection designs to ensure that comprehensive and meaningful insights could be derived from the dataset. Lastly, incomplete data on COVID-19 vaccine uptake hindered further analysis.

## 5 Future direction

The Implementation Network for Sharing Population Information from Research Entities (INSPIRE) (17), is actively engaged in extending the OMOP CDM to include data elements, particularly concepts, that are not currently captured by the existing ATHENA vocabulary set. This effort aims to improve the CDM's comprehensiveness and facilitate the integration of a broader range of data sources. The process of filling the gaps between the existing terminologies and concepts used for Africa-specific variables like ethnicity, housing conditions, socioeconomic status, etc., and the OMOP CDM vocabularies is ongoing.

Additionally, the INSPIRE team is collaborating closely with ministries across various African nations to secure access to COVID-19 datasets. These datasets will be processed and integrated into the OMOP CDM, enabling deeper analysis and comparisons on a pan-African scale. This initiative holds immense potential for uncovering regional trends, identifying areas of concern, and informing effective public health strategies, through the adoption of federated analytics where analysis algorithms are brought to the data, eliminating the need for physical data sharing.

## 6 Conclusion

The utilization of the OMOP CDM to harmonize COVID-19 sero-surveillance data from the Nairobi Urban HDSS has proven to be a good experience in mapping surveillance data into a standardized model for observational population health. It achieved data standardization which ensured consistency in data representation, enabling better analysis and data accessibility by adhering to a standard format, facilitating easier retrieval and utilization by different user groups, and opening opportunities for wider collaboration and knowledge sharing. The integration of population demographics with COVID-19 vaccination, and COVID-19 testing data into a standardized format provided a platform for improved analysis and interpretation of public health data. As we continue to work on COVID-19 data, the OMOP CDM will undoubtedly play a crucial role in standardizing it across studies for effective public health strategies. Population health data standardization in Africa is a catalyst for improving health outcomes. By addressing challenges and investing in standardization, African countries can build a robust health data ecosystem that enhances decision making, drives innovation, and strengthens the continent's readiness to tackle emerging health challenges.

## Data Availability

The datasets presented in this article are not readily available because the dataset is licensed and available according to the license agreement. Requests to access the datasets should be directed to the APHRC data portal at: https://microdataportal.aphrc.org/index.php/catalog/138.
